# Redefining Radiology: A Review of Artificial Intelligence Integration in Medical Imaging

**DOI:** 10.3390/diagnostics13172760

**Published:** 2023-08-25

**Authors:** Reabal Najjar

**Affiliations:** Canberra Health Services, Australian Capital Territory 2605, Australia; reabal.najjar@act.gov.au

**Keywords:** medical imaging, radiology, artificial intelligence, machine learning, deep learning, convolutional neural networks, computer-aided diagnosis, radiomics

## Abstract

This comprehensive review unfolds a detailed narrative of Artificial Intelligence (AI) making its foray into radiology, a move that is catalysing transformational shifts in the healthcare landscape. It traces the evolution of radiology, from the initial discovery of X-rays to the application of machine learning and deep learning in modern medical image analysis. The primary focus of this review is to shed light on AI applications in radiology, elucidating their seminal roles in image segmentation, computer-aided diagnosis, predictive analytics, and workflow optimisation. A spotlight is cast on the profound impact of AI on diagnostic processes, personalised medicine, and clinical workflows, with empirical evidence derived from a series of case studies across multiple medical disciplines. However, the integration of AI in radiology is not devoid of challenges. The review ventures into the labyrinth of obstacles that are inherent to AI-driven radiology—data quality, the ’black box’ enigma, infrastructural and technical complexities, as well as ethical implications. Peering into the future, the review contends that the road ahead for AI in radiology is paved with promising opportunities. It advocates for continuous research, embracing avant-garde imaging technologies, and fostering robust collaborations between radiologists and AI developers. The conclusion underlines the role of AI as a catalyst for change in radiology, a stance that is firmly rooted in sustained innovation, dynamic partnerships, and a steadfast commitment to ethical responsibility.

## 1. Introduction

Radiology, since its inception, has experienced a revolutionary journey, punctuating modern medicine with its profound influence. From the discovery of X-rays to the subsequent integration of artificial intelligence (AI) and machine learning (ML), this multifaceted discipline continually evolves, transforming itself and the healthcare ecosystem it underpins.

This comprehensive review scrutinises the interplay of AI and ML in radiology, exploring their foundational principles, historical progression, practical applications, inherent challenges, and ethical dilemmas. By enriching understanding of AI and ML’s contributions to radiology, the review aims to foster insightful discussions among clinicians, researchers, and policymakers, ultimately shaping the field’s direction and enhancing patient outcomes. The exploration delves into the fundamental constructs of AI and ML, their growing influence in radiology, practical integration strategies, and illustrative case studies across various medical specialties. Further, it addresses challenges such as data quality, ethical concerns, and contemplates potential future directions in AI-driven radiology.

### 1.1. Radiology in Modern Medicine

Radiology, the medical discipline centred on the utilisation of imaging modalities to diagnose and treat diseases, has emerged as a cornerstone of contemporary medicine, forming an integral part of clinical practice. It extends beyond mere disease detection to encompass treatment guidance and ongoing disease management. Expertise in diagnostic modalities such as computed tomography (CT), magnetic resonance imaging (MRI), positron emission tomography (PET), ultrasound, and X-rays guide immediate clinical interventions, treatment monitoring, and chronicle a visual narrative of a patient’s health. The intricate insights into anatomical, physiological, and molecular disease processes provided by medical imaging have a significant impact on patient care, facilitating the tailoring of treatments, thereby improving therapeutic outcomes and minimising adverse effects [1,2,3].

Radiology serves as a crucial gear in the intricate machinery of interdisciplinary medical teams. Radiologists deliver precise, timely imaging reports, thus enhancing communication among various specialists and shaping crucial decisions, which contributes to a holistic, patient-focused healthcare approach [4]. As valued consultative partners, radiologists offer key insights into the choice and interpretation of suitable imaging studies, playing a significant role in radiation safety and dose management while their expertise elucidates the clinical picture, offering insights that can markedly influence patient management [5,6].

### 1.2. From Roentgen to Magnetic Fields: A Brief History

**Figure 1:**
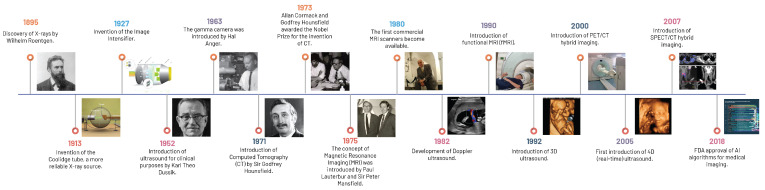
A historical timeline of the key discoveries in medical imaging.

The metamorphosis of modern medical imaging technology, from Wilhelm Roentgen’s pioneering discovery of X-ray technology in 1895 to contemporary advanced techniques, epitomises the relentless pursuit of scientific advancement and its profound impact on radiology (Figure 1). Roentgen’s unprecedented X-ray innovation offered a non-invasive glimpse into the human body, forming the foundation for modern imaging. Despite its initial limitations in 2D representation and soft tissue contrast, this fundamental concept laid the groundwork for more sophisticated, non-invasive imaging modalities [7].

The advent of CT in 1973 by Sir Godfrey Hounsfield and Allan Cormack marked a significant milestone, transcending the limitations of 2D imaging by introducing a three-dimensional (3D) format [8]. CT utilises the basic principle of differential absorption but couples this with the synchronised rotation of X-ray sources and detectors around the patient’s body, paired with sophisticated computational algorithms, enabling the reconstruction of 3D volumetric data from the collected 2D images [7].

Introduced in the 20th century, ultrasound imaging marked a departure away from ionising radiation-based technologies by using high-frequency sound waves to create real-time images of internal body structures. Its non-ionising radiation nature, real-time imaging capability, and cost-effectiveness have allowed broad applicability in various clinical fields, such as obstetrics, gynaecology, cardiology, and emergency medicine [9]. As an indispensable tool in emergency and critical care medicine, its transformative role, especially through point-of-care ultrasound (POCUS), has facilitated rapid bedside assessments and hastened clinical decision-making [7].

In the 1970s, Paul Lauterbur and Sir Peter Mansfield led the development of MRI, a technology that employs a robust magnetic field and radio waves to generate exceptionally detailed images of the body, particularly of soft tissue structures [10,11]. The non-ionising nature of MRI, coupled with its unrivalled soft tissue differentiation ability, revolutionised medical imaging. Manipulation of RF pulse sequence timing in MRI further enhanced its diagnostic utility, enabling the acquisition of various image types and discernment of distinct tissues and pathologies [7].

### 1.3. From Film to Function: An Evolution in Radiology

Parallel to the disruptive innovations in imaging modalities, another pivotal shift was occurring in the late 20th century: the evolution from film-based to digital radiography and the introduction of Picture Archiving and Communication Systems (PACS). This transition radically improved the efficiency of image acquisition, storage, and retrieval, while also enabling the seamless sharing and transfer of images within and across healthcare institutions [12].

The transformation in medical imaging technology did not stop with these innovations. Functional imaging techniques such as PET, which is uniquely characterised by its use of radiolabelled biochemical substances, and Single-Photon Emission Computed Tomography (SPECT), employing gamma-emitting radionuclides to trace biological processes, illuminated metabolic and biological processes, opening a window into cellular activity and providing invaluable insights into the functional status of organs [13,14].

3D imaging heralded a significant evolution in medical imaging by providing a more precise understanding of spatial relationships within the body, thereby improving diagnostic accuracy and surgical planning. The subsequent development of four-dimensional (4D) imaging further pushed the boundaries by incorporating the element of time, allowing for real-time monitoring of physiological processes [15].

The confluence of functional and anatomical imaging gave rise to hybrid imaging technologies such as PET/CT and SPECT/CT. These modalities amalgamate the strengths of both techniques, providing comprehensive diagnostic information. For instance, PET/CT combines the metabolic insight of PET with the detailed anatomical context of CT, considerably enhancing the accuracy of lesion localisation and characterisation [16].

Lastly, the emerging field of interventional radiology, which leverages imaging for guidance during minimally invasive procedures, has reshaped the healthcare landscape. By providing real-time visualisation of the target area, these procedures offer enhanced precision, potentially improving patient outcomes and reducing recovery times. For example, image-guided biopsies provide a safer and less invasive alternative to surgical biopsies, leading to fewer complications and shorter hospital stays [17].

### 1.4. A Glimpse into the Future: New Frontiers

Radiology’s future promises transformation through the integration of virtual/augmented reality (VR/AR) and AI, heralding a new era of medical imaging. Originating from the gaming and entertainment industries, VR/AR technologies are gradually permeating radiology, providing an immersive environment advantageous for radiology training and clinical practice. In the latter, the technologies can augment imaging data visualisation, thereby enhancing diagnosis and treatment planning [18].

AI, particularly its subset machine learning, is radically improving radiology, strengthening image analysis, and mitigating diagnostic errors. AI algorithms process and interpret data, performing tasks that emulate or even surpass human cognitive capabilities. ML, through exposure to labeled examples, is capable of extracting complex, high-level data, even from unlabelled datasets. By integrating AI into VR/AR technologies, the potential to boost radiological efficiency, improve diagnostic accuracy, and improve treatment planning exponentially exists [19].

Over the last two decades, the radiology community has refined computer-aided diagnosis (CAD) tools based on ML, which are set to bring about an integrated diagnostic service by incorporating radiology, pathology, and genomics data to improve the performance of CAD and enhance the productivity of radiology service by AI-assisted workflow [20].

Notwithstanding, the integration of AI and VR/AR in radiology encounters technical hurdles, notably incorporating AI-derived results into existing workflows. A proposed roadmap, however, advocates for AI-based image analysis algorithm integration, featuring a radiologist-AI feedback loop system for continuous improvement. This is exemplified by a case study demonstrating improved detection of brain metastases with AI application and radiologist feedback [21].

The ethical, legal, and societal implications of these technologies in radiology warrant careful scrutiny. The application of AI in radiology, while promising, can raise ethical issues, particularly related to bias and “black box” issues (i.e., the lack of transparency in AI decision-making processes). Advocacy for ethical AI underscores the need to address possible discriminatory effects and injustices, recommending future radiology-focused AI developments to incorporate social science perspectives [22].

## 2. The Fundamentals of Artificial Intelligence and Machine Learning

This section offers a journey through complex developments of AI and ML, sketching their historical trajectory and explaining the distinctive yet interconnected terminologies of AI, ML, and Deep Learning (DL). It also sheds light on the key ML algorithms and techniques that have shaped the technological landscape, underscoring the indelible imprint they have left on it.

### 2.1. Chronicle of Artificial Intelligence: Milestones and Breakthroughs

**Figure 2:**
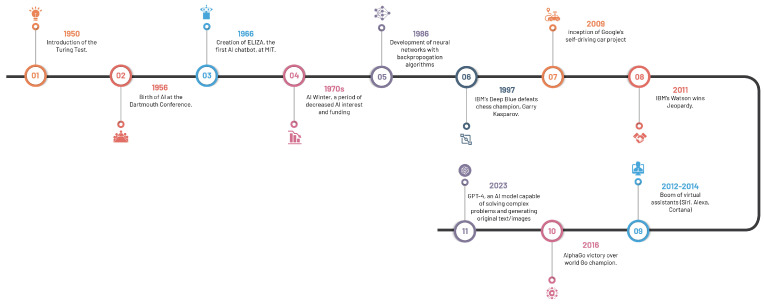
Significant milestones in the evolution of artificial intelligence.

The compelling development of AI, spanning from antiquity—marked by folklore and tales of artificial beings—to our current era of sophisticated AI systems, has roots in the philosophical depictions of human cognition as a mechanistic process (Figure 2). The inception of modern AI concepts is largely accredited to the development of the programmable digital computer in the 1940s, reaching a milestone with the field’s official establishment at the 1956 Dartmouth Conference [23].

The 1970s saw a significant leap forward with the emergence of rule-based expert systems like MYCIN, a seminal creation by Buchanan and Shortliffe [23,24]. These expert systems, designed to replicate human expertise by leveraging knowledge bases and inference engines, laid the foundation for AI’s influential role in medical diagnosis and clinical decision-making.

Machine learning algorithms, which surfaced in subsequent years, spearheaded a novel paradigm for data-driven predictions and classification. Decision trees introduced in 1986, support vector machines in 1995, and neural networks in 1986, collectively broadened the AI horizon in healthcare [25,26,27]. These algorithms propelled the analysis of extensive datasets, thereby inaugurating a novel epoch of pattern recognition and predictive modelling in healthcare.

The turn of the century witnessed a paradigm shift with the advent of deep learning approaches, particularly convolutional neural networks (CNNs). Surpassing previous methodologies in image recognition tasks, CNNs, architecturally modelled on structure and function of the human brain, precipitated major advancements in medical image classification, segmentation, and detection, due to their capacity to learn hierarchical representations from vast amounts of labelled data [28,29].

The accelerated progression of AI in recent years is a by-product of the synergistic interplay of two factors: the surge in big data and advancements in computational power. The widespread dissemination of electronic health records (EHRs), medical imaging archives, and annotated datasets provide ample training data, while progress in hardware, including graphical processing units (GPUs) and distributed computing, have expedited the deployment of computationally demanding AI algorithms [30].

The rapid advancement of AI language models, such as GPT-4, has led to unique applications and implications across a multitude of discplines, including healthcare and medical imaging [31]. These models, whilst capable of generating human-like text and facilitating communication, have raised salient concerns. The potential for these models to contribute substantially to medical research and patient care is undeniable; however, experts express reservations about their limitations and potential to inadvertently engender inequities or disseminate misinformation, thus emphasising the need for robust strategies to manage these risks responsibly. These strategies include increasing transparency about potential harms, promoting early detection of issues, and implementing regulatory measures and peer reviews [32]. By doing so, the goal is to ensure that AI technologies, including language models, are utilised optimally and ethically, contributing positively to medical imaging and healthcare outcomes.

By recognising these milestones, we can appreciate the progression of AI in healthcare, particularly in medical imaging. The intertwining of rule-based systems, traditional machine learning algorithms, and the transformative influence of deep learning techniques has laid the foundation for the current state-of-the-art AI applications in radiology and other medical specialties.

### 2.2. Decoding the Terminology: AI, ML, and DL

**Figure 3:**
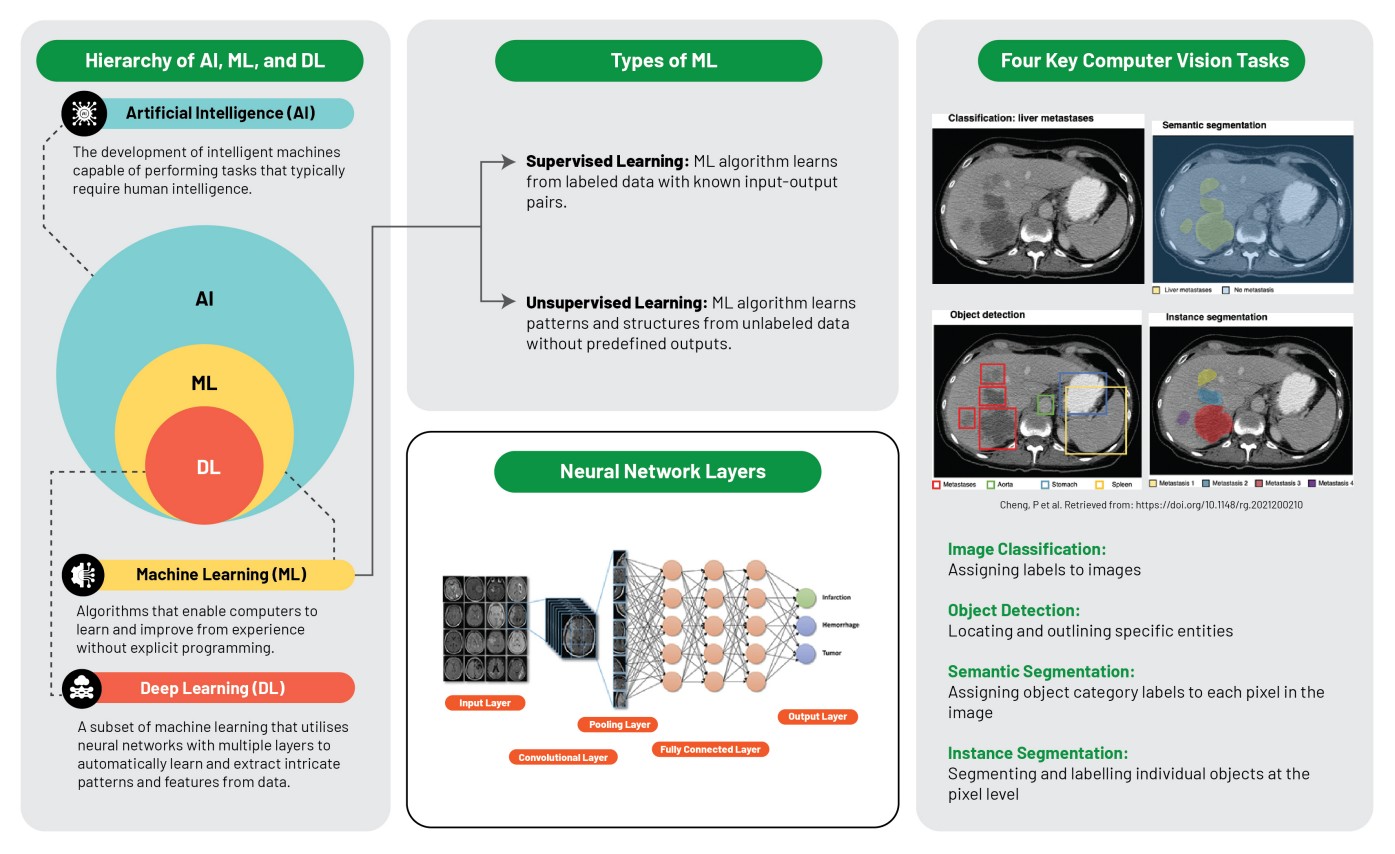
Schematic illustration of artificial intelligence and machine learning frameworks [35].

The computational intelligence landscape is an expansive system, constituted of distinct yet interconnected systems, with each sector playing a specific role and interacts uniquely within the data science sphere (Figure 3).

Artificial intelligence is defined as the replication of human intelligence in machines that are programmed to emulate human cognition and actions, encompassing learning, problem-solving, reasoning, and perception. AI can be classified into two major types: narrow AI, which is designed for specific tasks (e.g., facial recognition or voice commands), and general AI, which mimics a broader spectrum of human intellect. AI aims to develop systems with autonomous intelligent functionality, capable of problem-solving, decision-making, and performing tasks typically requiring human intelligence [33].

Machine learning, a subset of AI, is centred around the development of software that learns autonomously from accessed data. The learning process is derived from the analysis of observations or data to identify patterns and make informed future decisions based on these observations. Its fundamental objective is to enable computers to adapt their actions autonomously without human intervention. The primary categories of ML algorithms are supervised learning, which predicts or classifies new data based on examples, and unsupervised learning, which identifies inherent patterns and structures within the data without guidance from pre-established outputs [34].

Deep learning, an ML subset, utilises multilayered artificial neural networks (aptly coined “deep”), enabling DL algorithms to model and understand complex data patterns. These algorithms are especially effective for tasks where manual feature extraction proves challenging, such as image or speech recognition. It is noteworthy that these feature layers are data-learned, not human-engineered, embodying an architecture inspired by the human brain and have proven successful in visual recognition tasks, even outperforming human performance [28].

Recognising the nuanced interrelationships between AI, ML, and DL aids in conceptualising each subfield’s contribution and progression within the broader AI narrative. AI lays the foundation for ML and DL. ML amplifies AI’s potential by enabling machine data learning, and DL further deepens these capabilities with neural networks that decipher complex data patterns. Each field enriches the wider AI domain, culminating in the modern AI landscape where each layer contributes to the evolution of intelligent systems.

### 2.3. Machine Learning Foundations: Algorithms and Techniques

Machine learning is anchored by a plethora of algorithms and techniques that enable computers to learn from data. The two primary types of machine learning, supervised and unsupervised learning, lie at the heart of this field.

Supervised learning, a dominant machine learning variant, harnesses pre-established examples or training data, consisting of input-output pairs. The objective is to formulate a function that maps input data to corresponding outputs, enabling accurate predictions or classifications for unfamiliar data. Central to supervised learning are algorithms such as linear regression, logistic regression, and decision trees [36].

In contrast, unsupervised learning navigates the data space autonomously to uncover inherent patterns, structures, or relationships devoid of predefined outputs. Its focus is on discovering intrinsic data structures, thus offering insights that could potentially solve complex problems. Notable unsupervised learning algorithms include clustering techniques like k-means, hierarchical clustering, and dimensionality reduction methods such as principal component analysis (PCA) [37].

Artificial Neural Networks (ANNs) simulate the operational framework of the human brain, executing intricate tasks via a network of interconnected artificial neurons arranged in layers. The backpropagation algorithm, a vital cog in ANN operations, exhibits high fault tolerance, ensuring the system’s functionality despite occasional neuronal failures [38]. ANNs facilitate feature extraction and sophisticated pattern recognition, crucial for machine learning, which enhance data representation or class differentiation by aiding raw data pre-processing for feature extraction or selection [39].

Developments in ANNs have given rise to complex structures like deep learning models comprising multilayer neurons. Notably, CNNs employ convolution in lieu of standard matrix multiplication in certain layers. Tailored for processing pixel data, CNNs excel in tasks related to pattern recognition in images, audio, or text, substantially contributing to computer vision and Natural Language Processing (NLP) by simplifying complex patterns into abstract representations through layers of features [40].

## 3. Integrating AI into Medical Imaging: The Dawn of Radiology 2.0

This section delves into the remarkable role of AI within medical imaging, spotlighting the revolutionary shifts it catalyses in the world of radiology. Its multifaceted potential, from revolutionising image acquisition to reshaping radiological analyses, and from streamlining reporting to crafting personalised medical narratives, positions AI at the epicentre of the ongoing healthcare revolution. Beyond radiology, this transformation extends to other areas of healthcare—such as pathology, cardiology, genomics, drug discovery, and healthcare delivery—where the impactful strides of AI are being increasingly recognised. Concluding this exploration is the emergent paradigm of AI-facilitated personalised medicine, underscoring a more proactive, patient-oriented, and holistic patient care approach.

### 3.1. A Paradigm Shift in Radiology

**Figure 4:**
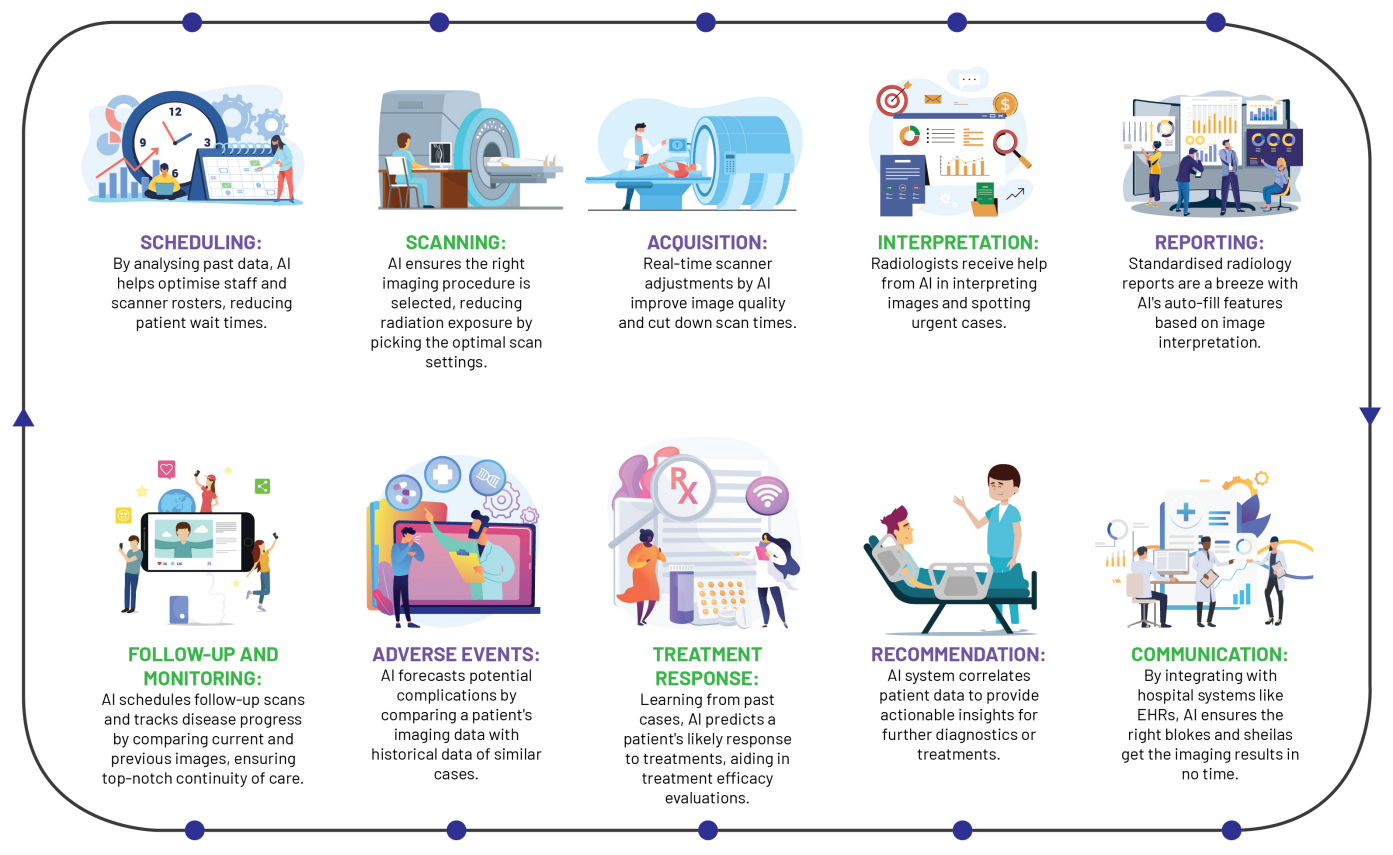
A streamlined workflow diagram illustrating the role of artificial intelligence in radiological practice.

AI has instigated a profound metamorphosis in the field of radiology, redefining traditional workflows and elevating the radiologist’s role (Figure 4). In the realm of image acquisition, AI augments scanning procedures, optimises image fidelity, and fosters sophisticated image reconstruction across MRI, CT, and PET modalities. Foremost among these advancements, deep learning accelerates MRI scanning, harmonising efficiency and quality, with commensurate progress witnessed in CT and PET image reconstruction [41].

AI significantly streamlines the acquisition of radiologist analyses on chest X-rays, as evidenced by a study wherein an AI system reduced interpretation delivery times from 11.2 days to a mere 2.7 days, reinforcing the potency of automated triaging systems in streamlining healthcare workflows and amplifying patient care standards [42].

As one of the pioneering healthcare specialties to adopt digital technology, radiology has capitalised on machine learning in CAD tools for over two decades, demonstrating robust performance in sensitivity and specificity [43]. Although clinical adoption has been slow due to various challenges, AI is posited as a pivotal tool to surmount these, enhancing CAD performance, streamlining radiology services, and fostering the development of integrated diagnostic services.

The integration of AI into radiology reporting has structured and annotated data to bolster report uniformity and streamlining patient history tracking. These cutting-edge tools generate comprehensive task lists, incorporating pertinent information from the patient’s history into EHRs, with the prime objective of enhancing report accessibility and integration into care pathways [44].

Beyond AI’s transformative influence on reporting and imaging procedures, these state-of-the-art systems play a pivotal role in maintaining continuity in provider communication and patient care, validating correlations between imaging diagnoses, radiological reports, and treatment plans, alerting providers to any discrepancies. Moreover, AI optimises personnel allocation and scanner usage and reduces radiation exposure, thereby boosting efficiency and quality of care [45]. By virtue of its broad-spectrum capabilities, AI is redefining the landscape of radiology, cementing its indispensable status within the discipline.

### 3.2. Beyond Radiology: Broader Applications of AI in Healthcare

AI has indelibly altered several healthcare areas, demonstrating its capability to enhance clinical practice beyond radiology via improvements in diagnostics, genomics, drug discovery, and healthcare delivery optimisation.

Pathology has witnessed the successful application of AI algorithms in tissue analysis, markedly enhancing diagnostic accuracy and speed. Automated image analysis tools enable pathologists to scrutinise tissues microscopically, identifying subtle histopathological attributes often overlooked by the human eye [46]. AI also accelerates the move towards digital pathology, converting traditional glass slides into digital scans for remote diagnostics and collaborative work—both of which are vital in the digital age of telemedicine [47].

AI shows considerable promise in cardiology, particularly in interpreting electrocardiograms and echocardiograms. Sophisticated ML algorithms detect complex cardiac patterns and abnormalities, accurately predicting conditions such as atrial fibrillation and myocardial infarction [48]. Its exponential growth in echocardiography is evident, with automated algorithms aiding in the interpretation of cardiac structure parameters, mitigating interobserver variability, and enhancing diagnostic precision [49].

The complex nature of genomics renders it an ideal candidate for AI intervention. DL techniques decipher genomic data, aiding in identifying genetic variants linked to disease susceptibility, and opening avenues for tailored treatment strategies based on individual genetic profiles [50].

AI has proven invaluable in the field of drug discovery by expediting the search for potent therapeutic compounds, and subsequently, accelerating the drug development process. For example, AI can predict the pharmacokinetic and pharmacodynamic properties of novel compounds, pinpoint potential drug targets, and simulate clinical trials, substantially reducing both time and costs associated with drug development [51].

Additionally, AI’s ability to optimise healthcare delivery is remarkable. AI-driven predictive analytics can enhance hospital workflows, accurately predicting patient admission rates, and optimising resource allocation [52]. AI applications in cost reduction have also emerged, with machine learning algorithms identifying inefficiencies in healthcare systems, thus enabling cost-effective care (SHAH 2021). The overarching aim of these AI applications is to improve patient outcomes by streamlining diagnostic processes and personalising treatment plans.

### 3.3. A New Era of Personalised Medicine

AI has catalysed a paradigm shift in the arena of personalised medicine. With its unparalleled capacity for processing vast amounts of complex data, AI is broadening the scope of personalised medicine, venturing into previously uncharted territories.

One of the most significant contributions of AI to personalised medicine is its potential to unlock the treasure trove of information embedded in EHRs. By harnessing the power of advanced machine learning algorithms, AI can discern patterns within EHRs, which can provide critical insights into specific disease states or risk factors. This process enables a proactive approach to patient care, allowing healthcare providers to foresee potential health risks and intervene accordingly [4]. In a landmark study by Rajkomar et al. (2018), AI was successfully utilised to predict medical events using data from EHRs, underlining its instrumental role in preventative medicine [53].

By integrating various sources of patient data—ranging from medical imaging and EHRs to genomic data—AI enables a more holistic approach to healthcare. This unification allows for personalised treatment plans, precise disease risk prediction, and improved monitoring of treatment responses. AI systems also enhance clinical decision-making by harmonising patient-specific data with up-to-date scientific discoveries, generating clinically relevant recommendations [54]. However, it is vital to ensure that AI’s implementation is patient-centric and ethical considerations such as data privacy, security, and algorithmic bias are rigorously adhered to. This vigilance guarantees the equitable application of AI technologies, respecting the rights and needs of all patients [4].

In conclusion, AI is shaping a new era of truly personalised patient care. However, it is paramount to acknowledge the necessity for continuous research and meticulous validation to ensure the safe and effective deployment of AI in healthcare. Multidisciplinary collaborations, involving specialists from AI, radiology, genomics, and clinical practice, are crucial for fine-tuning AI-driven models and technologies. Such partnerships will help maximise the potential benefits of AI, leading to enhanced patient outcomes and propelling the field of medicine into the future.

## 4. Practical Applications of AI in Radiology Practice

This section critically examines the practical applications of AI in the field of radiology, elaborating on the novel approaches it brings to imaging techniques, diagnosis, and patient care. By navigating through AI-driven methodologies like DL and CNNs, the section illuminates how AI is redefining the way image segmentation and classification, and diagnostics are conducted. Moreover, it explores the prognostic power of radiomics and predictive potential of AI in optimising workflows. Throughout the discourse, the section also confronts the inherent challenges and bottlenecks in the integration of AI within radiology, underpinning the critical need for interpretability, validation, standardisation, and the need to preserve the human element in healthcare.

### 4.1. Image Segmentation and Classification

DL has ignited a significant shift within radiology that is particularly noticeable in the domains of image segmentation and classification, where substantial strides have been made. The advancements brought about by these AI-centric methods have amplified the precision and speed of diagnosis, thereby amplifying the competency of radiologists and raising the bar of patient care. Nevertheless, the assimilation of AI brings about numerous challenges that need to be overcome to facilitate its optimal integration and application within radiology.

CNNs, given their inherent ability to learn complex patterns through backpropagation, have emerged as formidable tools for computational visual tasks, including various radiological applications. Their distinct architectural layers, from calculations in convolutional layers to the generation of predictions in fully connected layers, all converge to form a proficient object detection system. These networks have demonstrated remarkable proficiency in object detection tasks, thanks to their integrated capabilities of feature extraction, semantic segmentation, and the handling of multi-scale features. The efficiency and effectiveness of CNNs can be further amplified through the application of transfer learning, which allows for the reuse of pre-existing models. This, in turn, enhances accuracy, facilitates efficient training even with limited datasets, and minimises the need for labour-intensive and error-prone manual segmentation [40,55].

Illustrating CNNs’ potential, the segmentation of lung nodules from CT scans using AI has shown superior performance in the early detection and treatment of lung cancer, achieving an area under the receiver operating characteristic curve (AUROC) of 94.4% and outperforming six radiologists in the task [56]. Similarly, CNNs have played a pivotal role in the segmentation of brain tumours from MRI scans and the analysis of retinal images for early symptoms of diabetic retinopathy, further underlining AI’s broad applicability and versatility of AI in the sphere of medical imaging [40,55].

Image classification, an equally critical application of AI in radiology, leverages CNNs to differentiate between normal and pathological findings. AI models have been engineered to distinguish between benign and malignant tumours in mammography, achieving performance metrics comparable to human radiologists, thus facilitating early detection of breast cancer [57].

Notwithstanding these significant strides, integrating AI within radiology invites a suite of challenges that need to be addressed. The construction of robust and reliable AI models necessitates access to extensive datasets and thorough validation, both of which can be challenging due to privacy concerns and the heterogeneous nature of medical imaging data. Second, ensuring the interpretability and explicability of the “black-box” dilemma in deep learning models is paramount to garner trust and adoption from both radiologists and patients. Lastly, integrating AI into existing clinical workflows and fostering a harmonious human-AI collaboration are essential to fully realise the potential of AI in radiology and translate these technological advances into tangible enhancements in patient care.

### 4.2. Advancing Diagnostics with AI and CAD Systems

The third wave of AI, notably the integration of deep learning technology into CAD systems, has propelled radiology into a digital era. The emergence of AI-integrated CAD (AI-CAD) systems has revolutionised radiology services, underpinning transformative changes in the landscape of diagnostics. These systems have redefined how radiologists interpret images, increasing diagnostic accuracy, reducing false positives, and significantly improving workflow efficiencies.

AI-CAD systems’ merit lies in their substantial reduction of false positives, enhancing dependability in clinical settings. This is corroborated by a study comparing AI-CAD and traditional CAD software, where the AI system outperformed by decreasing the false-positive marks per image (FPPI) by a significant 69%. It specifically excelled in identifying microcalcifications and masses, reducing false positives by 83% and 56% respectively. Such reductions optimise radiologists’ efficiency, potentially cutting their case reading time by an estimated 17%, and mitigate socioeconomic issues like patients’ unnecessary emotional stress and financial burden [58].

Employing ensemble learning methodologies can hone CAD systems further, as evidenced by a recent study deploying a calibrated ensemble of deep learners for the task of detecting abnormalities in musculoskeletal radiographs. This ensemble model excelled over individual models, even outperforming expert radiologists in three out of the seven upper extremity anatomical regions (with an AUC of 0.93, Accuracy of 0.87, and Precision of 0.93). These results lend compelling support to the utility of the calibrated ensemble approach in identifying abnormalities in musculoskeletal X-rays [59].

AI has also shown remarkable promise in equalling, and in some cases surpassing, the performance of radiologists in breast screening through the deployment of AI algorithms for automated patient triage and predicting treatment outcomes—tasks that extend beyond human capabilities [60]. However, to fully exploit the benefits of AI in routine breast imaging fully, there’s a need to ensure sufficient data availability for testing and monitoring AI algorithms pre and post-integration into healthcare systems [60]. Yet, the dawn of a new AI epoch has presented a promising avenue that is anticipated to spur the next major transformation in radiology: the growth of radiomics—a field seeking to unify data from radiology, pathology, and genomics to offer a comprehensive diagnostic service [20].

### 4.3. Prognostics with Radiomics and Predictive Analytics

Radiomics is an emerging field within medicine, predicated on the extraction of high-dimensional data from radiological images and harbours immense potential for the landscape of medical diagnostics, prognosis, and the evaluation of disease response to treatment. However, radiomics still faces challenges, including the need for standardisation and validation to ensure reliable and reproducible outcomes. The main strength of radiomics lies in its ability to complement traditional clinical practice with precise, quantitative information, thereby revolutionising medical decision-making processes [61].

The rapid exponential growth of medical imaging data has fostered a conducive environment for the application of ML and data-driven science. Radiomics-based decision-support systems for precision diagnosis and treatment are poised to become integral tools in the armamentarium of modern medicine. This, however, is not to say that the journey of radiomics towards full clinical applicability is without challenges. The field currently grapples with a lack of standardisation and validation that are quintessential for ensuring reliable and reproducible outcomes [61,62].

In this milieu, the advent of AI presents promising avenues for overcoming these challenges and unlocking the full potential of radiomics. Through modelling high-dimensional data, AI-driven analytics enable accurate predictions pertaining to disease progression, treatment response, or patient survival. This significant stride offers clinicians an unprecedented wealth of information that vastly transcends the limitations of human perception. Particularly within oncology, radiomics has proven instrumental in identifying molecular phenotypes and lymph node metastases, appraising treatment response, and prognosticating disease survival [61].

It is crucial to acknowledge that the amalgamation of AI into radiomics remains in its infancy. To fully harness its potential in medical imaging, a concerted effort towards research and development is crucial. A key facet of this development will be the facilitation of large-scale data sharing, the establishment of standardised data collection protocols, clear evaluation criteria, and robust reporting guidelines. These elements are fundamental to the maturation and widespread adoption of radiomics as a discipline, opening the door to a new era of precision medicine.

### 4.4. Workflow Optimisation Using AI

AI is gaining traction in radiology, aiming to optimise workflows and enhance non-interpretative tasks’ efficacy. When coupled with NLP, AI can automate the triage of imaging studies, prioritising urgent cases based on the retrieval and analysis of key data from patients’ EHR. This expedites patient triage, radiology reporting, and managing incidental finding follow-ups [20].

AI significantly enhances the radiology process by automating triage and improving report generation. It swiftly sorts and prioritises radiological studies like CT scans and MRIs based on urgency, highlighting critical cases for immediate review. This automation aids in consistently detecting severe conditions such as stroke, haemorrhage, and malignancy, reducing errors. The use of AI, particularly NLP, in non-interpretative tasks alleviates tedious aspects of the workflow, potentially mitigating radiologist burnout [4].

Furthermore, AI enhances the generation and interpretation of radiology reports. Deep Learning algorithms address the limitations of traditional reporting, including fatigue-induced errors or inconsistencies due to varied expertise levels. They detect and characterise findings to improve consistency, facilitate standardised report creation, and reduce errors. This additional analysis layer streamlines the workflow and augments report clarity, significantly contributing to the quality of radiology services [63].

The incorporation of AI transcends the limits of purely diagnostic capabilities, profoundly amplifying interdisciplinary collaboration and patient-radiologist communication. AI platforms serve as a vital conduit that nurtures a collective understanding of imaging results among disparate healthcare professionals as they possess the potential to demystify the complexity of medical terminologies for patients. This transparency aids in establishing a strong rapport between the patient and the radiologist while also cultivating a higher degree of patient involvement in their personal health [61].

As strides are made towards AI integration in radiology, it’s crucial to recognise that as of 2021, only 30% of radiologists reported clinical AI use, with over 70% expressing reluctance to invest in AI. Many perceived AI as offering negligible benefits, hinting at the field of radiology being in the “trough of disillusionment” phase in the AI adoption process [64]. This disillusionment emanates from factors such as scepticism about AI performance and applicability, a perceived lack of necessity, inadequate workflows for efficient AI utilisation, and a dearth of scalable AI-supporting infrastructure.

To transition into the “slope of enlightenment”, the field must establish infrastructure that supports optimal AI functionality, involving the redefinition and disruption of existing systems such as image management and PACS for intelligent workflow orchestration [65]. Notwithstanding AI’s potential, the importance of preserving the human element in patient care cannot be overstated, underlining that while AI can augment the work of radiologists, it is not a substitute for the nuanced judgement and empathetic communication that are at the heart of patient care.

## 5. Case Studies: AI across Medical Specialties

### 5.1. Neuroradiology

**Figure 5:**
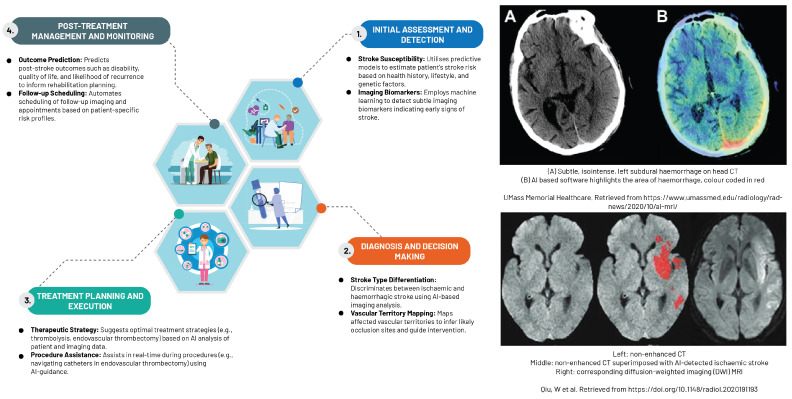
An overview of machine learning driven applications in neuroradiology [68,69].

The rapidly expanding field of ML, particularly supervised techniques and DL, has proven to be indispensable in managing high-dimensional data within the realm of neuroradiology (Figure 5). This cutting-edge technology has facilitated the early detection of a different stroke subtypes, as evidenced in the study by Yedavalli et al. (2021) [66]. CNNs demonstrate remarkable expertise in various tasks, including detecting infarcts or haemorrhages, segmentation, classification, and identification of large vessel occlusion. The application of CNNs in these areas has significantly influenced the approach to stroke treatment, as expounded by the research carried out by Soun et al. (2021) [67].

AI transcends diagnostic boundaries to bolster clinical decision-making, particularly in scenarios marked by substantial inter-rater variability. Its applications span from classifying stroke subtypes and detecting haemorrhages to identifying segmentation and large vessel occlusions. This advancement presents distinct advantages for facilities managing a small number of stroke patients or those functioning as regional hubs [70].

An ever-growing body of research underscores AI’s potential in bolstering decisions related to thrombolysis and thrombectomy. Shlobin et al. (2021), for instance, devised an AI model capable of precisely detecting large vessel occlusions using CT imaging, which demonstrates a high level of sensitivity and specificity in identifying patients suitable for timely thrombectomy intervention [71]. Zhu et al. (2022) utilised AI algorithms to predict thrombolysis responses in patients afflicted with acute ischaemic stroke, integrating imaging features with clinical data to support clinicians in formulating the most effective treatment strategies [72].

AI also plays a critical role in the early detection of neurodegenerative disorders, specifically for conditions like Alzheimer’s and Parkinson’s diseases. Sophisticated AI algorithms have been designed to analyse MR images for detecting specific biomarkers or characteristic patterns associated with these conditions. The task of discerning subtle changes in brain structure or function, which is crucial in diagnosing these diseases, is made more efficient with AI due to its ability to detect refined voxel-level patterns and provide objective, quantitative assessments [73].

Furthermore, AI has demonstrated its potential in predicting postoperative outcomes for brain and spine surgeries. By examining preoperative imaging data, AI models can generate prognostications regarding surgical outcomes, such as the likelihood of complications or the extent of functional improvement, aiding surgeons in treatment planning and managing patient expectations, as documented by Soun et al. (2021) [67].

### 5.2. Oncological Imaging

**Figure 6:**
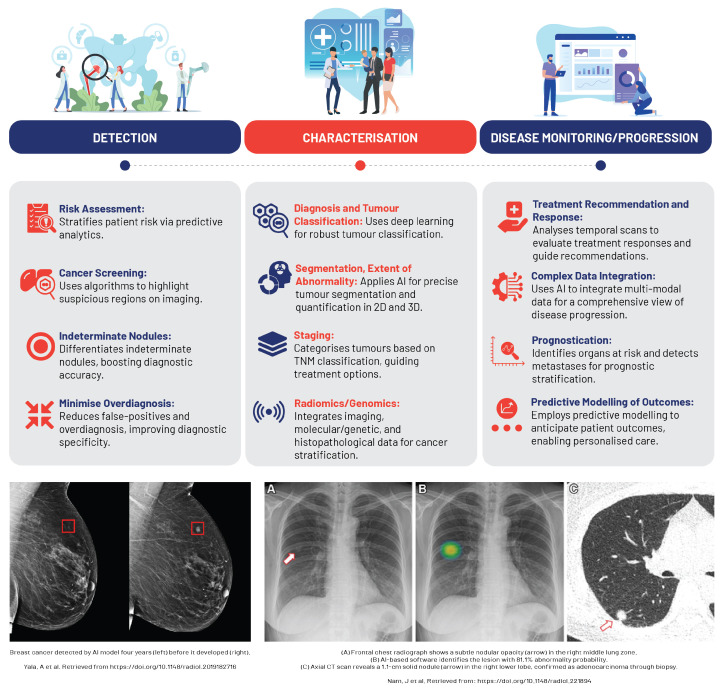
An overview of machine learning applications in oncological imaging [82,83].

AI and ML technologies, fuelled by the surge in high-performance computing, have catalysed significant advancements in oncology, particularly in cancer imaging (Figure 6). In precision oncology, the synergy between AI, superior computing, and deep learning strategies, coupled with the integration of multi-omics data, has streamlined cancer diagnosis, prognosis, and treatment [74,75].

The intrinsically digital nature of oncological imaging lends itself to AI and ML applications. Here, the imaging pipeline from acquisition to interpretation, reporting, and communication thrives within the digital space, enabling efficient data capture for AI and ML analysis. As a result, these technologies are being actively explored and adopted in cancer imaging, which constitutes a significant proportion of the workload in numerous healthcare facilities [74].

In tumour detection and classification, AI is increasingly being used to discern between benign and malignant lesions and various tumour types, particularly in diagnostics for breast, lung, and prostate cancers, with AI-based devices already making their way into clinical practice [75]. Studies reveal that deep learning models and CNNs can classify lung nodules on CT scans and differentiate renal cell carcinoma subtypes on MRI with high accuracy, often rivalling the expertise of experienced radiologists [76,77].

AI algorithms provide an objective, consistent means of assessing changes in tumour size or metabolic activity changes, automating measurements that were traditionally time-consuming and susceptible to inter-observer variability, such as those under the Response Evaluation Criteria In Solid Tumors (RECIST) [78]. AI accomplishes this by harnessing radiomic features—high-dimensional data extracted from radiological images—to build mathematical models adept at detecting subtle changes indicative of treatment response [4]. Furthermore, AI plays a key role in monitoring treatment response by quantifying tumour changes via granular analysis of medical image subunits (pixels/voxels). These smaller elements can be examined by computers to reveal objective mathematical features linked with disease behaviour or outcomes [74]. AI also procures valuable prognostic insights by analysing radiomic signatures, such as texture analysis, to predict survival rates in lung cancer patients from pre-treatment CT images, while radiomic features extracted from MRI scans have shown a correlation with recurrence risk in patients with glioblastoma [79,80]. Consequently, the integration of AI in radiology leads to efficient and precise tracking of tumour progression, significantly enhancing overall treatment assessment and patient care.

Traditional methods of monitoring radiation therapy response, based on manual assessment of changes in tumour size and characteristics, are often subjective and may overlook subtle indicators of treatment efficacy. AI, particularly CNNs, provide an objective means of assessing treatment response, utilising vast datasets of annotated imaging scans to accurately identify and delineate tumours [78]. This automation streamlines the planning process, potentially enhancing treatment outcomes via precise radiation dosing. The effectiveness of this approach is often quantified by the Sørensen–Dice coefficient (DSC), facilitating early and accurate therapy efficacy assessment and timely treatment adjustments, if necessary [81].

### 5.3. Cardiovascular Imaging

**Figure 7:**
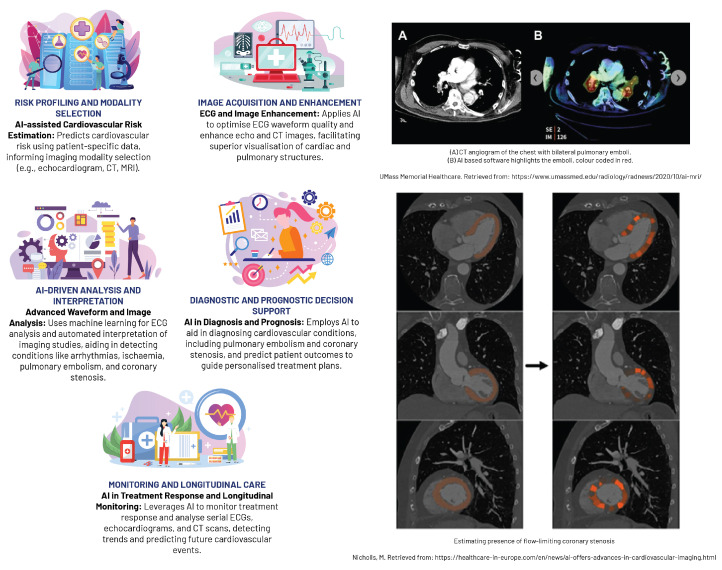
An overview of machine learning applications in chest imaging [68,86].

AI has considerably advanced cardiovascular imaging in recent years, enabling enhanced detection and quantification of heart diseases, comprehensive analysis of vascular abnormalities, and the integration of multi-modality imaging data (Figure 7). AI algorithms can efficiently interpret complex imaging data, recognising initial stages of cardiac diseases, such as coronary artery disease and congestive heart failure, through modalities like cardiac CT, MRI, or echocardiography. For example, ML models and CNNs have demonstrated the ability to automatically detect coronary artery calcification and perform automatic segmentation of left ventricular myocardium, respectively, with strong correlation to manual analyses [84,85]. AI technology has also enhanced the functional evaluation of the left ventricle in echocardiographic diagnosis by automating tasks traditionally reliant on visual observation and manual boundary tracing, such as measuring left ventricular ejection fraction using the Simpson method. Such advancements promise to reduce the dependence on physician experience, increasing the repeatability and accuracy of these evaluations [84].

Beyond cardiac strucuture conditions, AI is also instrumental in the analysis of vascular abnormalities, such as aortic aneurysms or peripheral artery disease, facilitating early intervention and potentially improving patient outcomes. CNNs have proven effective in evaluating abdominal aortic aneurysms from CT images, demonstrating high accuracy in detecting and sizing these potentially life-threatening conditions [87]. Furthermore, AI-assisted standard section recognition has significantly reduced the time required for evaluation, enhanced detection ability, and improved the accuracy of novice practitioners, which has been particularly beneficial in settings with limited resources for training echocardiography physicians [84].

A major breakthrough in AI-based cardiovascular imaging is the integration of data from multi-modality imaging, which combines information from CT, MRI, and echocardiography to provide a holistic representation of cardiac structure and function. This consolidated data is essential for complex assessments, such as detecting ischaemia or planning interventions. For instance, ML algorithms can merge perfusion data from MRI with coronary anatomy from CT, generating sophisticated 3D heart models, thus improving the detection of cardiac ischaemia and facilitate precise procedural planning [88,89].

### 5.4. Abdominal Imaging

**Figure 8:**
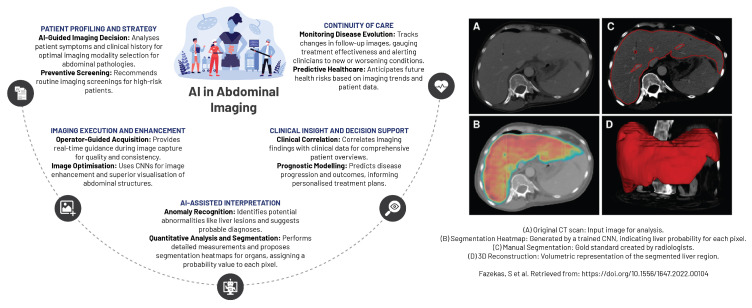
An overview of AI-driven applications in abdominal imaging [91].

AI has fostered unprecedented advancements in abdominal and pelvic imaging, particularly within the domain of gastrointestinal imaging (Figure 8). AI’s contributions have been pivotal in improving detection, diagnosis, and staging of liver and pancreatic diseases. The conceptualisation of diverse AI-based predictive models has broadened the diagnostic spectrum, encompassing gastrointestinal and inflammatory diseases, non-malignant conditions, and the detection of bowel bleeding using cutting-edge technology such as wireless capsule endoscopy [90]. AI has also proven instrumental in detecting hepatic-associated fibrosis by leveraging EHRs to derive meaningful insights into patient health data and medical history. Furthermore, AI’s fusion with endoscopic ultrasound technology has substantially improved both the accuracy and speed of diagnosing pancreatic carcinoma, consequently enhancing patient management strategies [90].

In the specialised fields of hepatology and pancreatology, the integration of AI with various imaging techniques has brought about a diagnostic paradigm shift in diagnosing liver and pancreatic diseases. The techniques that have benefited from AI include ultrasound, endoscopic ultrasonography, CT, MR, and PET/CT. AI’s influence permeates beyond diagnostics, aiding in the selection of the most appropriate diagnostic test for a patient based on individualised medical profile. Furthermore, AI has been instrumental in optimising image quality, accelerating image acquisition, and predicting patient prognosis as well as their response to treatments [92].

AI has reshaped abdominal and pelvic imaging with its ability to deliver precise and reproducible imaging diagnoses. The technology facilitates automated or semi-automated segmentation and registration of the liver and pancreatic glands, along with their associated lesions, thereby increasing the diagnostic accuracy and treatment efficacy. The integration of radiomics introduces novel quantitative metrics into radiological reports, enriching the detection and characterisation of focal lesions and diffuse diseases of the liver and pancreas, leading to potential enhancements in clinical outcomes [92].

In nephrology, contemporary AI applications display remarkable potential in anticipating the onset of acute kidney injury before significant biochemical changes become evident, potentially allowing for timely interventions to prevent disease progression. Additionally, AI’s ability to identify modifiable risk factors for chronic kidney disease progression offers valuable insights for preventative care [93].

Furthermore, in the sphere of renal tumour detection, AI models have demonstrated proficiency matching, or even exceeding human accuracy, in interpreting imaging studies. This impressive feat could augment prognostication and decision-making processes post-renal transplantation. The heightened precision in detecting and diagnosing renal tumours fosters more effective treatment strategies, potentially leading to improved patient outcomes [93].

## 6. Challenges, Limitations, and Future Directions

**Figure 9:**
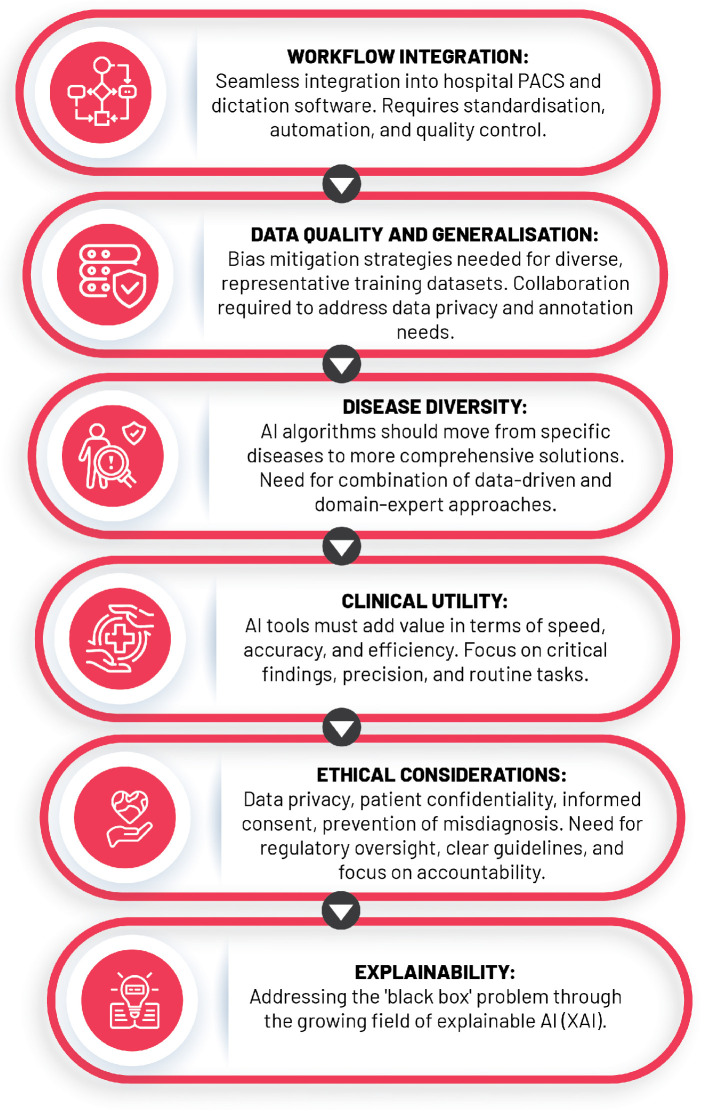
Challenges of AI integration into clinical practice.

The expansion of AI in healthcare, particularly in the field of diagnostic radiology, has presented unprecedented opportunities to enhance the quality and efficiency of patient care. Despite this, the rapid growth comes with a myriad of challenges, including the necessity for adequate data quality and volume, the “black box” dilemma, integration into clinical practice, and ethical considerations. This section dissects these issues and proposes potential resolutions that could facilitate the assimilation and responsible usage of AI in radiology while addressing various technical, infrastructural, regulatory, and human factors (Figure 9).

### 6.1. Data Quality, Quantity, and the “Black Box” Problem

The performance of AI algorithms, essentially mathematical mirroring of reality, depends not only on their training datasets and precision but also on calibration in interpreting medical images depends on comprehensive datasets that accurately reflect varied patient demographics, including age, sex, ethnicity, and disease stages [94,95].

The construction of such datasets is frequently obstructed by representation biases due to the utilisation of restricted demographic groups or specific clinical settings [96]. Strategies like data augmentation, oversampling, and undersampling are often employed to remedy data scarcity, ensuring dataset diversity and balanced representation during model training [97].

It is essential to recognise and address potential risks associated with biased or unrepresentative data as mismanagement can inadvertently perpetuate health disparities and yield AI models with subpar performance in certain patient populations. The “black box” problem in AI, denoting the lack of transparency in AI models, complicates error detection and bias identification, thus adversely affecting underrepresented groups and clinical utility [22]. Tackling these challenges requires a concerted effort to diversify data collection, combat bias in AI system design, perform population subgroup-based performance analyses, and use representative population samples for clinical validation [22].

Adding to these strategies, a growing field known as explainable artificial intelligence (XAI) seeks to demystify AI decision-making processes through improving inference reliability and enhancing transparency and interpretability [98]. XAI techniques, such as saliency maps, feature importance, and surrogate models, aid in visualising and explaining the rationale behind AI models’ decisions, making them accessible to both experts and lay audiences [99,100].

### 6.2. Clinical Integration of AI into Radiology Practice

The integration of AI into clinical radiology has been met with both enthusiasm and scepticism, necessitating a clear roadmap to address the multifaceted challenges that arise. Central to these is the prerequisite for robust hardware and reliable software, which are instrumental in managing the vast amounts of data generated by medical imaging systems. The untapped potential in the estimated 97% of unused hospital data can be harnessed by AI-enabled applications, substantially augmenting disease trajectory prediction and treatment regimen modification [101].

The effective implementation of AI demands high-performance hardware capable of executing complex computations in real time, implying the need for significant investment in powerful, reliable hardware infrastructure to circumvent any adverse effects on patient care due to system failures [101]. On the other hand, the software must be robust, intuitive, and designed to integrate within existing radiological systems, necessitating close collaboration between AI developers, radiologists, and other healthcare professionals [102]. AI also holds the promise of alleviating administrative burdens that beleaguer radiologists who, on average, dedicate approximately 16.6% of their working hours (roughly nine hours per week) to administrative tasks [103].

Navigating the hurdles of regulatory approval and ongoing monitoring is equally imperative, as AI tools need to undergo rigorous validation to demonstrate safety and effectiveness before gaining regulatory approval [104]. Once approved, continuous monitoring and evaluation are necessary to track performance and ensure continuous improvement and the delivery of reliable results. Lastly, successful AI integration into radiology hinges on acceptance and adoption by end-users, primarily radiologists [103]. This highlights the importance of providing sufficient training and support to ensure radiologists can effectively use AI tools and comfortably integrate them into daily practice.

### 6.3. The Ethical Conundrums

The ethical implications of AI integration within radiology unearths several ethical quandaries, namely data privacy and security, patient confidentiality, informed consent, misdiagnosis risks, and the preservation of the human element in patient care.

The intersection of data privacy and security with AI pivots on the governance of patient data, notably when considering public-private partnerships involving large tech corporations. A review of commercial healthcare AI unveiled that a considerable fraction of these technologies are under the control of private entities, raising alarms about potential data misuse. This is epitomised by the DeepMind incident, which involved the transfer of patient data from the United Kingdom to the United States without explicit patient consent [105]. These events call for more stringent regulatory oversight to guarantee that patient data remains within its original jurisdiction and is safeguarded against unauthorised access.

Patient confidentiality and informed consent, inextricably linked to data privacy, require patients to exercise autonomy over their data, comprehending its use, potential risks, and associated benefits. This aspect becomes especially significant within AI-driven healthcare, where the black box nature of learning algorithms can veil the decision-making processes [105]. Consequently, requisite safeguards and transparency procedures need implementation to uphold privacy and secure patient autonomy.

Furthermore, the potential for misdiagnosis, liability, and accountability in AI-assisted radiology raises significant ethical concerns. Although AI offers promising diagnostic capabilities, it is susceptible to errors and biases, which can lead to incorrect diagnoses and patient harm [106]. Addressing this issue necessitates the development and implementation of clear guidelines and policies for AI use in medical settings, with an emphasis on decision-making accountability and delineating the responsibilities of healthcare professionals and AI systems.

With that being said, the risk of over-reliance on AI emphasises the importance of preserving the human element in patient care. While AI can enhance healthcare delivery and complement the skills of healthcare professionals, it should not overshadow the irreplaceable expertise and nuanced decision-making inherent in the practice of medicine, particularly given the complexity and variability of individual cases [22]. As the role of AI in radiology continues to evolve, these ethical considerations must remain at the forefront of discussions, policy-making, and research, ensuring the responsible and equitable application of this transformative technology in healthcare.

### 6.4. Bridging the Gap: Collaboration between Radiologists and AI Developers

The growing intersection of AI and radiology necessitates a synergistic collaboration between radiologists and AI developers to create advanced tools that drive clinical innovation and improve patient care. This section discusses the fusion of these disparate fields and the importance of interdisciplinary collaboration, its benefits, and strategies to bridge the gap between academia and industry.

Radiologists bring to the table an in-depth understanding of clinical needs, disease processes, and imaging interpretation nuances, while AI developers possess the technical acumen to design, implement, and optimise machine learning algorithms. This diverse expertise is not just complementary but also essential for the development of AI tools that are clinically viable, safe, and effective. Without the input of radiologists, AI tools risk being designed without consideration for real-world clinical workflows, limiting their utility or potentially compromising patient safety. Conversely, without the technical skills of AI developers, radiologists would struggle to leverage the vast potential of AI in imaging analysis [65].

Interdisciplinary research is the key to marrying these distinct skill sets. Collaboration between radiologists and AI developers can result in AI tools more attuned with clinical needs, effectively bridging the gap between theory and practice. Such partnerships also encourage a collective sense of ownership and responsibility, driving the adoption and optimisation of AI tools in clinical settings. A common model for fostering collaboration is the Academia-Industry Collaboration Plan (AICP), which outlines processes and methods for establishing partnerships between academia and industry [107]. This is crucial as universities supply the workforce and innovative ideas, while the industry provides necessary funding for research and innovation.

However, promoting collaboration between academia and industry is not without its challenges. One significant hurdle that can stifle innovation and delay the deployment of AI tools in radiology is the contemporary dichotomy between these two sectors. While academia often focuses on theory and exploration, industry is more concerned with practical applications and market viability [107]. To overcome this, there are several strategies that can be employed. First, joint projects involving both academic and industry partners can facilitate the exchange of ideas and resources, leading to innovative and robust AI solutions. Shared datasets enable a more diverse range of research, enhancing the generalisability of AI tools. Finally, open-source software offers a platform for collaboration, promoting transparency and reproducibility, both of which are fundamental to scientific progress.

### 6.5. Medical Education and Training in Healthcare

The incorporation of AI into clinical practice necessitates the acquisition of new skills. With AI algorithms capable of processing large volumes of data far beyond human capacities, the emphasis on memorising extensive medical information or developing procedural skills through repetitive practice may diminish. This shift necessitates the need for clinicians to acquire new skills, such as data science, statistics, and AI ethics, which will be crucial for interacting safely and effectively with AI technologies [108]. These skills will empower clinicians to competently input data, interpret algorithmic outputs, and communicate AI-derived treatment plans to patients.

In the face of this shift, the roles and responsibilities of radiologists may extend beyond image interpretation to encompass a broader understanding and application of AI technologies. Radiologists, traditionally concerned primarily with image interpretation, may find themselves more engaged in tasks such as AI model development, validation, or monitoring. For instance, a significant component of AI in radiology involves the labelling of images, a labor-intensive and costly process that demands the expertise of radiologists [20]. As AI technologies advance, radiologists will increasingly interact with decision support systems that can suggest diagnoses, alert test results, and even automate clinical documentation [108].

The professional evolution of radiologists may also coincide with significant scientific and technical advancements in radiomics and pathomics, facilitating the integration of diagnostic services and personalised medicine, especially in resource-poor settings with limited infrastructure where the adoption of AI might be challenging [20].

The Royal Australian and New Zealand College of Radiologists (RANZCR) has adopted a progressive approach to these challenges and opportunities. In their 2023 curriculum, AI-related topics have been incorporated, reflecting the growing recognition of the importance of AI in radiology and the need for radiologists to acquire new competencies. This crucial inclusion of AI ensures that RANZCR remains at the forefront of technology and innovation, solidifying its commitment to equipping radiologists with the most advanced tools and knowledge in the rapidly evolving field of medical imaging [109].

## 7. Conclusions

This review concludes by summarising the key insights, transformative potential, and future path of the intricate relationship between AI and medical imaging. With AI playing a pivotal role in modern radiology, it provides a plethora of advantages such as improved diagnostic accuracy, workflow efficiency, and personalised patient care. These advancements, including tools for image partitioning, categorisation, computer-aided diagnosis, and innovative diagnostic and prognostic tools driven by radiomics and predictive analytics, herald a promising potential for improving patient outcomes. However, challenges concerning data privacy, security, and the ‘black box’ nature of AI models remain to be addressed. Despite these hurdles, the future is promising with new algorithms and architectures broadening the scope of medical image analysis. An essential synergy between radiologists and AI developers is needed to foster interdisciplinary research and bridge academia and industry. This need also extends to preparing healthcare professionals for an AI-infused landscape and evolving the role of radiologists in the AI era. Embracing AI’s potential in redefining radiology involves not only an unwavering dedication to innovation and development of advanced algorithms, but also nurturing collaborations among radiologists, AI developers, patients, and policy-makers. These joint efforts should aim to meet clinical needs, translate research into practical applications, and ensure ethical AI deployment, always prioritising patient safety, privacy, and dignity. In this light, the forthcoming aeon of AI in radiology, though challenging, unfolds its vast potential in transforming healthcare.

## Data Availability

No new data were created or analysed in this study. Data sharing is not applicable to this article.
